# Current Strategies for Healthy Aging: The Interplay Between Nutrition, Metabolism, and Exercise

**DOI:** 10.7759/cureus.96848

**Published:** 2025-11-14

**Authors:** Ana Matos, Cezara Tihon, Carolina Costa, Catarina Domingues

**Affiliations:** 1 Family Health Unit Vale do Vouga, Entre Douro e Vouga Local Health Unit, São João da Madeira, PRT

**Keywords:** aging, exercise, neurodegeneration, nutrition, sarcopenia

## Abstract

Healthy behaviors, such as a balanced diet, physical activity, and health care, have favored an increase in life expectancy. However, in Western society and some developing nations, a sedentary lifestyle and poor nutritional choices are more common, increasing the risk of developing disease in old age. We propose to characterize the interplay between nutrition, metabolism, and exercise for better and worse: its contribution to a pathological state when unbalanced, while integrated dietary interventions and exercise promote metabolic health and healthy aging. Caloric-restricted diets or diets rich in compounds with antioxidant action may have a positive impact on aging in the nervous system, being a protective factor against cognitive decline. In addition to the beneficial effect of physical exercise on motor skills, fragility, and the prevention of sarcopenia, regular physical activity is also neuroprotective. An adequate diet and the practice of exercise are critical players for healthy aging, optimizing cognitive function, maintaining muscle mass, and a balanced metabolism.

## Introduction and background

It is well known that the percentage of the population classified as elderly will increase dramatically in almost all countries in the coming decades. A significant increase in average life expectancy, along with the improvement in socioeconomic conditions, coupled with a shift in the main causes of disease, has contributed to the growing global incidence of aging-related diseases. Indeed, many people spend more of their later years in poor health, with increased morbidity and greater health costs. Aging, due to the accumulation of damage and the impairment of repair mechanisms, is associated with a gradual biological process of physiological decline, characterized by subtle changes in cellular homeostasis, particularly evident in tissues with high energetic demand [1]. Despite the increased knowledge on the cellular mechanisms associated with aging as discussed by López-Otín et al. - genomic instability, telomere attrition, epigenetic alterations, loss of proteostasis, disabled macroautophagy, deregulated nutrient-sensing, mitochondrial dysfunction, cellular senescence, stem cell exhaustion, altered intercellular communication, chronic inflammation, and dysbiosis [2] - it is still necessary to seek strategies for healthy aging.

Aging with its associated changes predisposes to a variety of diseases, including cardiovascular disease, sarcopenia, neurodegeneration, and type 2 diabetes mellitus (T2DM). Increased body fat and decreased muscle and bone mass are events associated with aging. Sarcopenia, an age-related disorder characterized by loss of skeletal muscle mass and function, is a risk factor for a multitude of adverse health outcomes [3]. Along with declining cognitive, visual, and auditory function, sleep disturbances, depression, and increased fatigue lead to a decline in physical function, significantly increasing the risk of functional disability and loss of autonomy [4]. Deficiencies in episodic or declarative memory, spatial learning, and working memory are manifestations of aging-associated cognitive decline [4].

A sedentary lifestyle is associated with increased risk of developing several chronic diseases, such as T2DM, cardiovascular diseases, obesity, and cancer. Physical exercise contributes to healthy aging and reduces the morbidity rates of various age-related diseases. Being an easily promotable and implementable activity, the importance of physical activity has been increasingly highlighted [5].

In addition to the beneficial effect of physical exercise on motor skills and fragility, several studies highlight that the lack of regular physical activity appears to play a role in the development of depression, dementia, and neurodegenerative diseases [4]. On the other hand, exercise has a positive impact on aging in the nervous system, being a protective factor against cognitive decline. Physical exercise has been linked to beneficial effects on various brain functions, such as appetite and stress regulation, mood, memory, and cognitive function, in addition to effects on various systems, such as the cardiovascular, endocrine, and musculoskeletal systems [6]. With advancing age, even healthy and capable individuals experience some decline in cognitive performance, making implementing an active lifestyle crucial for healthy aging. However, many elderly individuals do not develop a systematic exercise program. It is important to highlight that the socio-economic status may be an important factor influencing the regular practice of physical activity, but only a few studies have examined this association [7]. A study involving cross-sectional data of 1507 participants in Germany showed significant negative associations between self-reported physical activity and socioeconomic status for both men and women, while objectively measured physical activity was positively associated with socioeconomic status, significant in men but not in women [8].

## Review

Search strategy 

A critical analysis of the literature was carried out after a search in the Medline (Pubmed) database using combinations of the terms “aging”, “neurodegeneration”, “sarcopenia”, “exercise”, and “nutrition”, including articles from the last seven years, written in English with available abstracts. From the search, the selection was conducted first based on the article title and then, after reading the abstract. The references listed in recent and relevant articles retrieved through the search were also consulted.

Neurodegenerative diseases

Neurodegenerative diseases are a complex set of late-onset pathologies clinically characterized by decreased cognitive function and motor coordination, dyskinetic movements, and irreversible changes in behavior and personality. Examples include Parkinson's disease (PD), Alzheimer's disease (AD), Huntington's disease (HD), and Amyotrophic Lateral Sclerosis. Depending on the region affected by progressive degeneration and/or neuronal death, the clinical forms and symptoms associated with these pathologies differ [4]. With a multifactorial etiology, the interaction between genetic susceptibility and environmental risk factors has been identified as a source of influence for the progressive development of neurodegenerative changes. Indeed, human cognitive function is influenced by several factors, including genetics, lifestyle, nutrition, disease, trauma, exposure to xenobiotics, and both normal and pathological aging. Adopting health-promoting behaviors in early middle age may be beneficial in reducing the rate of cognitive decline and the risk of dementia [6], as demonstrated by the practice of physical activity and a balanced diet over 20 years [9]. However, longitudinal data regarding the long‐term impact of initiating exercise in late life in older adults is still limited. Comparing older adults who initiated routine, sustained exercise with inactive older adults shows an important longitudinal trajectory with exercise protecting from age‐related declines in physical performance, opposingly to cumulative deficits across strength, aerobic endurance, and mobility in those who remained sedentary [10].

Mitochondrial Dysfunction and Neurodegeneration

Neurons are a highly specialized cell population with a high dependence on adenosine triphosphate (ATP), resulting in a correlation between cognitive decline, mitochondrial alterations, and brain hypometabolism [11,12]. Although the intrinsic cellular heterogeneity of brain tissue raises the difficulty of the study of metabolic alterations occurring in the brain, age-dependent alterations of neuronal metabolism are now believed to occur prior to neurodegeneration. Mitochondrial dysfunction has been consistently recognized as an important factor in the pathogenesis of neurodegenerative diseases, such as Parkinson’s (PD) and Alzheimer’s disease (AD), with contributions from oxidative stress, mitochondrial DNA mutations, ATP depletion, calcium dysregulation, and apoptosis induction [13].

High levels of oxidative stress-induced mutations have been detected in mitochondrial DNA in the brains of elderly individuals, as well as in the brains of Parkinson's and Alzheimer's patients [14-17]. Mitochondrial dysfunction affects metabolism globally, with decreased glucose uptake detected in AD, which correlates with cognitive decline [18,19]. Additionally, reduced levels of GLUT1 (glucose transporter in astrocytes) and GLUT3 (glucose transporter in neurons) in AD also appear to contribute to hypometabolism and neurodegeneration [20]. Furthermore, changes in mitochondrial number and morphology, as well as changes in bioenergetics, are systemic mitochondrial alterations identified in AD [21]. The accumulation of β-amyloid causes mitochondrial dysfunction in AD. β-amyloid inhibits the activity of mitochondrial complexes II and IV, leading to decreased ATP levels and increased reactive oxygen species (ROS) formation [22]. Mutations in several genes encoding proteins that affect mitochondrial homeostasis are highly penetrant mutations that result in rare monogenic forms of PD, characterized by mitochondrial dysfunction, oxidative damage, and apoptosis [23]. Decreased content and activity of complex I of the electron transport chain have been detected in PD [24,25]. Epidemiological evidence has suggested an association between exposure to pesticides, such as paraquat (complex I inhibitors of the electron transport chain), and an increased risk of developing PD [26]. It has also been shown that type 2 diabetes increases iron concentration in several deep gray matter structures in elderly individuals, causing neurotoxicity [27].

Benefits of a healthy lifestyle

Although considerable progress has been made in understanding the cell death processes associated with neurodegenerative diseases, allowing the design of new therapeutic strategies, there is still no highly effective treatment. Neurodegenerative diseases have a significant impact on the quality of life of patients, with serious social, psychological, and economic burdens for individuals and their families. Several studies suggest that lifestyle changes can be made at any age to reduce the risk of developing neurodegenerative changes or, at an early stage, protect the brain from irreversible damage. Examples of lifestyle choices that can help reduce the risk of neurodegenerative disease include regular physical activity, a healthy diet, a regular sleep pattern, and cognitive training [28-34].

Changes in sleep duration and quality are frequently observed in older adults. These changes can affect cognitive performance and thus influence the development of certain types of dementia [35]. Melatonin, which has known effects in the treatment of sleep disorders, has a neuroprotective effect in AD and PD, reducing inflammation and the accumulation of protein aggregates [36]. The circadian rhythm is crucial in regulating human physiology, such as sleep-wake cycles, metabolism, hormone balance, and cognitive behavior. Disruption of this system, which may be caused by aging, lifestyle factors, and environmental influences, has been associated with reduced melatonin secretion, accumulation of beta-amyloid, contributing to neuronal dysfunction and cognitive decline [37,38]. A study of the association between circadian function, aging, and preclinical AD pathology in cognitively normal adults with a mean age of 66.6 years showed rest-activity rhythm fragmentation, independent of age or sex, highlighting that circadian dysfunction occurs very early in the course of AD and precedes the onset of cognitive symptoms [39].

A disrupted circadian rhythm, marked by age-related alterations such as decreased variation in sleep-wake patterns and instability in the timing of these patterns, can worsen age-related problems such as increased oxidative stress and inflammation [40]. Dysregulated gut microbiota rhythms and associated metabolic changes further enhance neuroinflammatory responses, increasing AD risk. Advancing age is accompanied by a chronic, progressive state of low-grade inflammation that is associated with an increased risk of developing age-related conditions such as diabetes, memory impairment, and progressive loss of brain volume [41, 42].

The beneficial effects of physical exercise have been reported both in independent older individuals and individuals with cognitive decline [43,44]. A systematic review found that low-intensity physical activity, such as walking, is associated with a reduced risk of dementia and AD [36], but resistance training or combination training has also shown positive effects regarding cognitive function [43-48]. It has been suggested that physical exercise may increase neurotrophic factors, synaptic connectivity, and reduce the loss of dopaminergic neurons, resulting in recovery of motor function in PD, decreased inflammation, and improved mitochondrial function [49,50]. These results indicate that physical exercise leads to the recovery of motor function through mechanisms that are still unknown. Additionally, the practice of physical exercise as a preventative activity against neurodegeneration may involve beneficial effects on cardiovascular function and stimulation of cerebral blood flow, anti-inflammatory effects, and benefits in diseases such as obesity and T2DM. Metabolic syndrome (impaired glucose tolerance, abdominal or central obesity, hypertension, and hypertriglyceridemia) and T2DM may be responsible for an increased risk of age-related cognitive decline, vascular dementia, and AD. A study that followed more than 1,400 individuals for more than two decades demonstrated an approximately two-fold increase in the risk of cognitive decline in middle-aged individuals who are overweight and with high blood pressure, a risk that increased to 6.2-fold with the combination of these factors [51]. Advanced glycation end products (AGEs) play a role in neuronal dysfunction associated with diabetes mellitus, and hyperglycemia increases the formation of AGEs that accumulate with age [52]. Antidiabetic drugs from the thiazolidinedione family, such as rosiglitazone, decrease AGE-mediated neurotoxicity and improve cognition in animal models and patients with AD [53].

Besides the type of exercise, the duration of exercise, the gender of participants, and other chronic somatic conditions are factors influencing the effect of exercise on cognition benefits [45]. Social interaction may also act as a factor influencing the positive effects of physical activity on healthy aging. Chronic loneliness, which is increasingly considered a public health epidemic, acts as chronic stress at the biobehavioral, neuroendocrine, and mitochondrial levels, and is implicated in metabolic diseases [54]. The barriers (fitness and health; motivation/interest; fear of falling/history of falling; environmental) and motivators (support from family and friends; social interaction; personal benefits; and outside facilities) to physical activity vary according to gender, age, functional ability, and geographical location [55].

Dietary Interventions

Intermittent fasting is a controversial, popular practice associated with weight loss that requires eating only for a set number of hours a day. Intermittent fasting may have other health benefits beyond weight loss, as it appears to induce adaptive responses in the brain that decrease inflammation [56]. Studies have shown that alternate-day fasting over 8 to 12 weeks, alternate-day fasting trials of a 3- to 12-week or whole-day fasting lasting 12 to 24 weeks lead to a reduction in cholesterol and triglycerides [57,58].

Calorie restriction may also have neuroprotective effects by modulating neurogenesis and synaptic plasticity, as seen in a transgenic model of AD in which 30% caloric restriction for 4 months significantly decreased hippocampal atrophy and caspase-3 activation [59,60]. In non-human primates, caloric restriction preserved brain volume and enhanced cognitive function in aged monkeys, delaying the onset of age-related diseases and improving overall health and longevity [61,62]. However, while controlled studies in model organisms demonstrate clear benefits, human studies on caloric restriction are delayed, particularly in the elderly, by potential risks such as the risk of inadequate intake of nutrients, the associated alterations in muscle and bone [63]. A two-year, randomized controlled trial for non-obese individuals showed that a 25% reduction in daily caloric intake led to significant improvements in insulin sensitivity, lipid profile, inflammatory markers, and muscle function [64]. 

Carbohydrate-restricted diets, besides the impact on body weight, are linked to the readjustment of glycemic and insulin control as well as decreased inflammation. The ketogenic diet (KD) is a high-fat, low-carbohydrate, and moderate-protein intake diet that leads to metabolic changes in which ketone bodies, such as beta-hydroxybutyrate, are an alternate source of energy. Besides increased satiety, KD and the associated ketone bodies have been associated with neuroprotection [65-67]. However, small sample sizes and short study durations limit the conclusions on the diet's benefits.

Given the beneficial effects of antioxidants and the known critical role of oxidative stress in neuronal dysfunction, there has been growing interest in developing antioxidant therapies with the potential to increase the responsiveness of the physiological defense system. Cognitive decline appears to be associated with increased and prolonged formation of pro-inflammatory cytokines, even in healthy aging [68]. A recent study showed that elderly individuals have elevated levels of pro-inflammatory cytokines associated with reduced gray matter, with smaller volumes of lateral prefrontal cortex and hippocampus, and a higher risk of cognitive decline, particularly in inactive elderly individuals [69], supporting that with aging, a pro-inflammatory state develops in the periphery, but also in the central nervous system (CNS).

One strategy is a healthy dietary pattern rich in compounds with antioxidant action, such as vitamins A, E, and C, flavonoids, phenolic acids, and carotenoids [70]. The Mediterranean diet (MD), widely recognized for its health benefits, is characterized by high intake of fruits, vegetables, whole grains, fish, and olive oil. Numerous epidemiological studies support a neuroprotective action of the MD since adherence to the Mediterranean diet correlates with better cognition in elderly populations and a lower risk of cognitive decline and dementia [71,72]. Several studies have found that functional factors such as polyphenols, polysaccharides, unsaturated fatty acids, melatonin, and caffeine have positive effects with significant improvements in cognition and memory [73,74]. Unsaturated fatty acids inhibit β-amyloid production and Tau protein phosphorylation and reduce neuroinflammation, and melatonin has been shown to protect nerve cells and improve cognitive function by regulating mitochondrial homeostasis and autophagy. Several antioxidants capable of crossing the blood-brain barrier, such as vitamins E and C, are known to be effective against oxidative stress-mediated neuronal death and dementia. Although in vitro and animal model studies point to the neuroprotective potential of vitamin E [75], some clinical studies have not supported this potential in preventing dementia [76]. However, a clinical study showed that administration of vitamin C and/or E supplements resulted in a decreased risk of cognitive decline in people over 65 years of age [77], although at higher doses (≥400 IU/d) vitamin E supplements may increase all-cause mortality [78]. Polyphenols exhibit antioxidant, anti-inflammatory, and neuroprotective effects. Evaluation of the effects of oleocanthal from extra-virgin olive oil on β-amyloid burden in preclinical models of AD showed the anti-inflammatory and neuroprotective effects of biophenols from extra-virgin olive oil [79]. Modulation of the neurotrophin pathway is another mechanism that explains the neuroprotective action of polyphenol-rich diets since neurotrophic factors are essential for neuronal function and synaptic plasticity [80].

**Figure 1:**
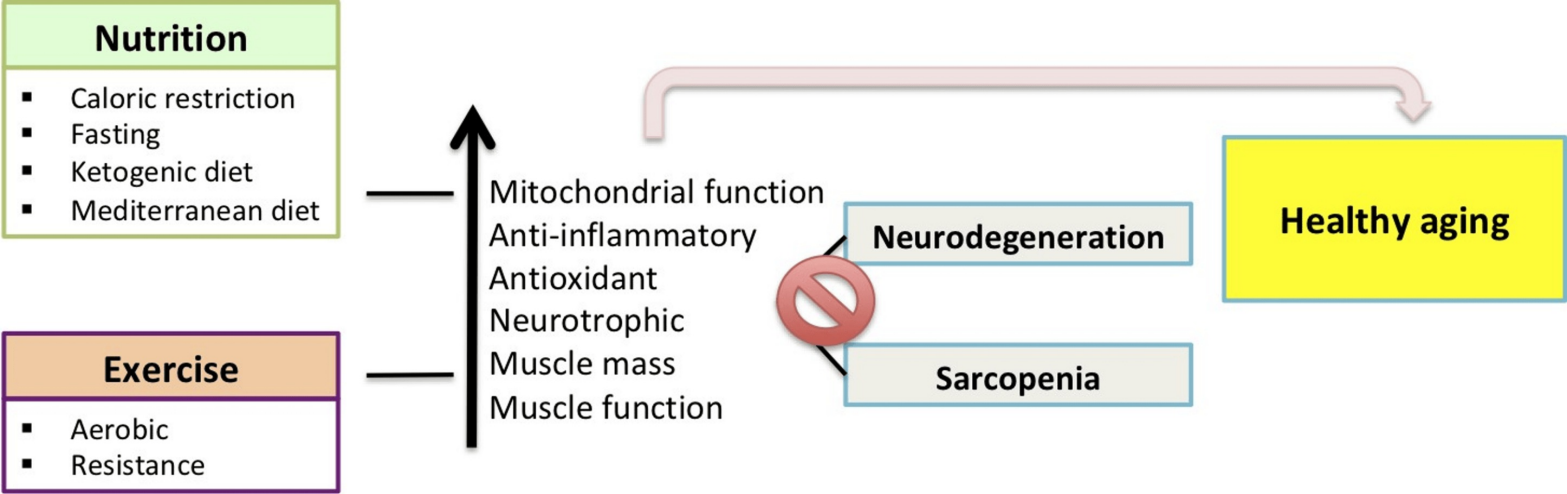
The interplay between nutrition and exercise for healthy aging The interplay between nutrition and exercise for healthy aging: its contribution to a pathological state when unbalanced, while integrated dietary interventions and exercise promote metabolic health and healthy aging. Poor dietary choices and a sedentary lifestyle have a profound impact on life expectancy and quality, contributing to the accumulation of damage and the development of aging-related pathologies such as cardiovascular disease, dementia, and type 2 diabetes mellitus (T2DM). A chronic state of unbalanced metabolism and oxidative stress contributes not only to the development and progression of T2DM and increased dementia risk but also impairs skeletal muscle health. This results in a vicious cycle in which impaired muscle health influences the development and progression of T2DM as well as the decline in cognitive function, while T2DM and neurodegeneration contribute to reduced muscle use and loss of skeletal muscle mass and function. Figure source: Original work of the authors.

Flavonoids are a large family of polyphenolic compounds found in fruits, vegetables, cocoa, and beverages such as wine and tea. Its neuroprotective action involves antioxidant capacity (glutathione synthesis and increased antioxidant protein content) and inhibition of apoptotic death [81]. Several studies also show that the intake of foods rich in flavonoids increases cerebral blood perfusion, resulting in acute improvements in cognition and increased markers of synaptic plasticity in animal studies [82]. Non-flavonoid antioxidants like curcumin are also neuroprotective. In a transgenic model of AD, curcumin inhibited neuroinflammation, prevented the development of amyloid aggregates, tau hyperphosphorylation, and memory impairments [83]. Co-administration of curcumin and melatonin, which also has antioxidant action, in a transgenic model of AD decreased inflammation and synaptic alterations, amyloid accumulation in the hippocampus and cortex, and induced increased levels of complexes I, II, and IV of the electron transport chain [84]. The effect of caffeine on cognitive decline and dementia in adulthood was analyzed in a systematic review, highlighting a potential correlation between moderate consumption of coffee or caffeine-rich beverages and a reduction in cognitive decline [85]. The beneficial action of caffeine has been shown in several neurodegenerative diseases, involving antioxidant, anti-inflammatory, and anti-apoptotic effects as well as neurotrophic effects [72,86,87]. Future research should further explore the mechanisms of action of these functional factors and develop relevant functional foods or nutritional supplements to provide new strategies and support for the prevention and treatment of neurodegenerative diseases (Figure 1).

Besides nutritional content, an important factor that influences the potential benefits of dietary patterns is the amount of ultra-processed foods with high levels of processed sugars and fats, contributing to an inflammatory environment and promoting neuroinflammation and altering brain function. A study’s two-decade follow-up period revealed that consumption of significant amounts of ultra-processed foods (sugar-sweetened beverages and processed meats, such as hot dogs, sausages, and deli meat) was 10% more likely to die, particularly from heart disease and diabetes [88].

Sarcopenia

Sarcopenia, highly prevalent in aged individuals as well as in obese individuals, is characterized by loss of muscle mass and function and is now considered a disease on its own [89]. Aging associated with a lack of regular physical activity contributes to an abnormal localization of fat in the skeletal muscle, which alters the metabolism of the skeletal muscle [3,90]. Skeletal muscle is notable for its ability to maintain glucose homeostasis by using insulin to mediate glucose disposal. Therefore, besides the impact on the quality of life, loss of skeletal muscle and function has profound effects on glucose metabolism and insulin resistance. T2DM, with increased prevalence in elderly individuals, is also associated with decreased glucose metabolism by the skeletal muscle, favoring muscular atrophy. Insulin resistance, mitochondrial dysfunction, and oxidative stress associated with T2DM accelerate the decline of muscle mass and function [91,92]. Alterations in mitochondrial metabolism caused by mitochondrial DNA deletions that accumulate with aging favor cell death in muscle fibers [93].

Protective Role of Different Types of Exercises

The mechanisms underlying the health benefits of regular physical activity involve multiple adaptive pathways in several tissues. Several studies in recent years have demonstrated the preventive effect of regular exercise against the typical effects of aging and the promotion of brain health, resulting in functional adaptation and improved performance on specific tasks. Comparing sedentary older adults with older adults who practiced aerobic exercise throughout their lives, it was found that regular aerobic exercise preserves the microstructural integrity of white matter, which may be related to motor control and coordination in older adults [94]. A longitudinal study with 716 elderly individuals without dementia for 4 years demonstrated that higher levels of physical activity reduce the risk of developing AD [79]. Practice of aerobic exercise for 3 months results in cognitive benefits related to complex object recognition, positively correlated with vascular perfusion and increased hippocampal volume in healthy adults aged 60 to 77 years [95]. Since older adults are not functionally, cognitively, or metabolically homogeneous, there is variability in the levels of physical activity required to broadly optimize health and musculoskeletal function. Improvement of cognitive function after aerobic exercise, resistance training, a multi-component training program, tai chi, and yoga was observed regardless of baseline cognitive status and the type of physical exercise practiced, in adults over 50 years of age [96,97].

Despite growing evidence of the beneficial effects of regular exercise on cognition, the mechanisms underlying the association between exercise, structural and neurochemical changes, and cognition are not fully understood. These beneficial effects on cognitive health have been associated with the stimulation of neurotrophic signaling, adult hippocampal neurogenesis, synaptogenesis, and synaptic plasticity [98]. Studies conducted in animal models but also in humans support the relationship between structural and functional changes in the brain, prevention of cognitive decline through exercise, and metabolic changes essential for the homeostasis of neuronal function and improvements in cerebral vascular perfusion [98-101]. It was recently demonstrated in mice that treadmill training for 5 weeks prevented the decrease in the number of neural stem cells due to aging and stimulated neuronal differentiation [101]. In an animal model of Alzheimer's, 10 weeks of treadmill training induced beneficial effects on learning-related tasks, an effect associated with increased dendritic complexity in the hippocampus and amygdala [102]. Bolz and collaborators observed that mice that practiced voluntary physical activity through free access to the wheel for 11 days showed an increase in the number and length of dendrites in newly formed neurons, facilitating synaptic contact and processing at the hippocampal level, with better results in terms of pattern distinction and object recognition [103]. In addition to structural observations such as the prevention of hippocampal atrophy, several studies point to anti-inflammatory and antioxidant effects promoted by physical activity related to the modulation of anti-inflammatory cytokine profiles, redox-sensitive transcription factors, and antioxidant enzymes [104]. These potentially have beneficial effects on neuroplasticity, cellular excitability, modulate the expression of growth factors, and consequently maintain and stimulate neuronal function.

Regarding sarcopenia, while the practice of aerobic exercise improves cardiorespiratory fitness and promotes energy expenditure, resistance exercise is a powerful stimulus for the musculoskeletal system, with the consequent development of muscular strength and endurance. However, the impact of resistance training on muscle morphology and mobility is still under discussion [105-107]. A comprehensive training program combining aerobic and resistance exercise is recommended, even for older adults, ranging from five sessions of 30 minutes of moderate-intensity aerobic exercise each week to three sessions of 20 minutes of vigorous-intensity training [108]. The applications of these guidelines must recognize the diversity in older adults and the influence of the physical and functional capacity of the individual and chronic conditions that affect the ability to safely practice exercise. Sarcopenia can predispose individuals to falls, fractures, hospitalization, and mortality. Sarcopenia is associated with a significantly higher risk of mortality, independent of population and sarcopenia definition, since it can predispose individuals to falls, fractures, and hospitalization, significant loss of quality of life, and considerable healthcare expenditure [109].

Limitations

Despite the general acceptance of the positive impact of a balanced diet and physical activity on healthy aging, different dietary and physical activity baselines of participants in studies are a major factor influencing the success of these strategies on healthy aging. Furthermore, genetic and metabolic heterogeneity are also factors influencing the outcome. Cultural environmental and psychological characteristics of the participants are key factors that affect the adherence of the participants in chronic studies. Furthermore, socioeconomic status, social contact, and other factors also importantly influence aging, and in many studies, these variants are not adequately explored and their role valued.

## Conclusions

The rise of age-related pathologies and their significant impact on the quality of life of patients, as well as the social, psychological, and economic burdens for the affected families, is a strong argument for the sensitization regarding strategies to prevent the onset of age-related diseases and improve human health. One key participant in the aging process is oxidative stress, with increasing evidence supporting the therapeutic effects of the antioxidant action of polyphenols. A chronic state of unbalanced metabolism and oxidative stress contributes not only to the development and progression of T2DM and increased dementia risk but also impairs skeletal muscle health. This results in a vicious cycle in which impaired muscle health influences the development and progression of T2DM as well as the decline in cognitive function, while T2DM and neurodegeneration contribute to reduced muscle use and loss of skeletal muscle mass and function. Simple lifestyle changes that include an adequate diet and the practice of exercise must be implemented at any age.

## References

[1] Amorim JA, Coppotelli G, Rolo AP, Palmeira CM, Ross JM, Sinclair DA (2022). Mitochondrial and metabolic dysfunction in ageing and age-related diseases. Nat Rev Endocrinol.

[2] López-Otín C, Blasco MA, Partridge L, Serrano M, Kroemer G (2023). Hallmarks of aging: an expanding universe. Cell.

[3] Thompson LV (2025). Prevention of sarcopenia. Adv Exp Med Biol.

[4] Dugger BN, Dickson DW (2017). Pathology of neurodegenerative diseases. Cold Spring Harb Perspect Biol.

[5] Gbessemehlan A, Proust-Lima C, Letenneur L, Amieva H, Pérès K (2025). Healthy aging: how does a multidimensional construct of functional ability predict objective and subjective outcomes?. BMC Geriatr.

[6] Clare L, Wu YT, Teale JC, MacLeod C, Matthews F, Brayne C, Woods B (2017). Potentially modifiable lifestyle factors, cognitive reserve, and cognitive function in later life: a cross-sectional study. PLoS Med.

[7] Malkowski OS, Harvey J, Townsend NP, Kelson MJ, Foster CE, Western MJ (2025). Enablers and barriers to physical activity among older adults of low socio-economic status: a systematic review of qualitative literature. Int J Behav Nutr Phys Act.

[8] Stalling I, Albrecht BM, Foettinger L, Recke C, Bammann K (2022). Associations between socioeconomic status and physical activity among older adults: cross-sectional results from the OUTDOOR ACTIVE study. BMC Geriatr.

[9] Cadar D, Pikhart H, Mishra G, Stephen A, Kuh D, Richards M (2012). The role of lifestyle behaviors on 20-year cognitive decline. J Aging Res.

[10] Manning KM, Hall KS, Sloane R (2024). Longitudinal analysis of physical function in older adults: the effects of physical inactivity and exercise training. Aging Cell.

[11] Islam MT (2017). Oxidative stress and mitochondrial dysfunction-linked neurodegenerative disorders. Neurol Res.

[12] Watts ME, Pocock R, Claudianos C (2018). Brain energy and oxygen metabolism: emerging role in normal function and disease. Front Mol Neurosci.

[13] Guo C, Sun L, Chen X, Zhang D (2013). Oxidative stress, mitochondrial damage and neurodegenerative diseases. Neural Regen Res.

[14] Dölle C, Flønes I, Nido GS (2016). Defective mitochondrial DNA homeostasis in the substantia nigra in Parkinson disease. Nat Commun.

[15] Arthur CR, Morton SL, Dunham LD, Keeney PM, Bennett JP Jr (2009). Parkinson's disease brain mitochondria have impaired respirasome assembly, age-related increases in distribution of oxidative damage to mtDNA and no differences in heteroplasmic mtDNA mutation abundance. Mol Neurodegener.

[16] Mecocci P, MacGarvey U, Beal MF (1994). Oxidative damage to mitochondrial DNA is increased in Alzheimer's disease. Ann Neurol.

[17] Wang J, Xiong S, Xie C, Markesbery WR, Lovell MA (2005). Increased oxidative damage in nuclear and mitochondrial DNA in Alzheimer's disease. J Neurochem.

[18] Minoshima S, Giordani B, Berent S, Frey KA, Foster NL, Kuhl DE (1997). Metabolic reduction in the posterior cingulate cortex in very early Alzheimer's disease. Ann Neurol.

[19] Mosconi L (2005). Brain glucose metabolism in the early and specific diagnosis of Alzheimer's disease. FDG-PET studies in MCI and AD. Eur J Nucl Med Mol Imaging.

[20] Ding F, Yao J, Rettberg JR, Chen S, Brinton RD (2013). Early decline in glucose transport and metabolism precedes shift to ketogenic system in female aging and Alzheimer's mouse brain: implication for bioenergetic intervention. PLoS One.

[21] Perez Ortiz JM, Swerdlow RH (2019). Mitochondrial dysfunction in Alzheimer's disease: role in pathogenesis and novel therapeutic opportunities. Br J Pharmacol.

[22] Rhein V, Song X, Wiesner A (2009). Amyloid-beta and tau synergistically impair the oxidative phosphorylation system in triple transgenic Alzheimer's disease mice. Proc Natl Acad Sci U S A.

[23] Abou-Sleiman PM, Muqit MM, Wood NW (2006). Expanding insights of mitochondrial dysfunction in Parkinson's disease. Nat Rev Neurosci.

[24] Parker WD Jr, Parks JK, Swerdlow RH (2008). Complex I deficiency in Parkinson's disease frontal cortex. Brain Res.

[25] Haas RH, Nasirian F, Nakano K, Ward D, Pay M, Hill R, Shults CW (1995). Low platelet mitochondrial complex I and complex II/III activity in early untreated Parkinson's disease. Ann Neurol.

[26] Freire C, Koifman S (2012). Pesticide exposure and Parkinson's disease: epidemiological evidence of association. Neurotoxicology.

[27] Li J, Zhang Q, Che Y, Zhang N, Guo L (2021). Iron deposition characteristics of deep gray matter in elderly individuals in the community revealed by quantitative susceptibility mapping and multiple factor analysis. Front Aging Neurosci.

[28] Topiwala H, Terrera GM, Stirland L, Saunderson K, Russ TC, Dozier MF, Ritchie CW (2018). Lifestyle and neurodegeneration in midlife as expressed on functional magnetic resonance imaging: a systematic review. Alzheimers Dement (N Y).

[29] Dhaka P, Neha Neha, Kumar R, Hossain CM, Parvez S (2025). The interplay between circadian rhythms and aging: molecular mechanisms and therapeutic strategies. Biogerontology.

[30] Szychowska A, Drygas W (2022). Physical activity as a determinant of successful aging: a narrative review article. Aging Clin Exp Res.

[31] Lin FV, Tao Y, Chen Q, Anthony M, Zhang Z, Tadin D, Heffner KL (2020). Processing speed and attention training modifies autonomic flexibility: a mechanistic intervention study. Neuroimage.

[32] Styliadis C, Kartsidis P, Paraskevopoulos E, Ioannides AA, Bamidis PD (2015). Neuroplastic effects of combined computerized physical and cognitive training in elderly individuals at risk for dementia: an eLORETA controlled study on resting states. Neural Plast.

[33] Nolan E, Sun Y, Shi H (2025). The association between poor sleep health and Alzheimer's disease structural neuroimaging biomarkers. Alzheimers Dement.

[34] Shannon OM, Ranson JM, Gregory S (2023). Mediterranean diet adherence is associated with lower dementia risk, independent of genetic predisposition: findings from the UK Biobank prospective cohort study. BMC Med.

[35] Hafycz JM, Naidoo NN (2019). Sleep, aging, and cellular health: aged-related changes in sleep and protein homeostasis converge in neurodegenerative diseases. Front Aging Neurosci.

[36] Cardinali DP (2019). Melatonin: clinical perspectives in neurodegeneration. Front Endocrinol (Lausanne).

[37] Singh J, Kumar D, Kaur J, Singh A (2025). The rhythm of decline: circadian disruption in neurodegeneration. J Food Drug Anal.

[38] Eckhardt JL, Isenberg L, Aslanyan V (2025). Circadian rhythms are associated with higher amyloid-β and tau and poorer cognition in older adults. Brain Commun.

[39] Musiek ES, Bhimasani M, Zangrilli MA, Morris JC, Holtzman DM, Ju YS (2018). Circadian rest-activity pattern changes in aging and preclinical alzheimer disease. JAMA Neurol.

[40] Nagayach A, Bhaskar R, Ghosh S (2025). Interplay between circadian rhythm, ageing and neurodegenerative disorder. Open Biol.

[41] Andonian BJ, Hippensteel JA, Abuabara K (2025). Inflammation and aging-related disease: a transdisciplinary inflammaging framework. Geroscience.

[42] Zhang W, Sun HS, Wang X, Dumont AS, Liu Q (2024). Cellular senescence, DNA damage, and neuroinflammation in the aging brain. Trends Neurosci.

[43] Lin CC, Kao SC, Chueh TY, Hung TM (2025). The effects of chronic exercise interventions on executive function in healthy older adults and optimal training characteristics: a systematic review based on randomized controlled trials. Psychol Sport Exerc.

[44] Chang JY, Chou KR, Chang YL, Lin WY, Chiu HL, Liao YC, Yang CT (2025). Enhancing cognitive function and well-being in older adults with cognitive and physical decline: a meta-analysis of randomized controlled trials examining physical activity interventions. J Phys Act Health.

[45] Cui MY, Lin Y, Sheng JY, Zhang X, Cui RJ (2018). Exercise intervention associated with cognitive improvement in Alzheimer's disease. Neural Plast.

[46] Akalp K, Ferreira JP, Soares CM, Ribeiro MJ, Teixeira AM (2024). The effects of different types of exercises on cognition in older persons with mild cognitive impairment: a systematic review and meta-analysis. Arch Gerontol Geriatr.

[47] Qiu C, Han J, Xie Y (2025). Comparison of the efficacy of different exercise modes on MCI adults: a network meta-analysis. Brain Behav.

[48] Deng Z, Zeng D, Zhang Y, Jia D, Huang X (2025). Comparative effectiveness of multicomponent exercise interventions on cognitive function in people with cognitive impairmsent: a systematic review and network meta-analysis. J Clin Nurs.

[49] Palasz E, Niewiadomski W, Gasiorowska A, Wysocka A, Stepniewska A, Niewiadomska G (2019). Exercise-induced neuroprotection and recovery of motor function in animal models of Parkinson's disease. Front Neurol.

[50] Hou L, Chen W, Liu X, Qiao D, Zhou FM (2017). Exercise-induced neuroprotection of the nigrostriatal dopamine system in Parkinson's disease. Front Aging Neurosci.

[51] Feinkohl I, Lachmann G, Brockhaus WR (2018). Association of obesity, diabetes and hypertension with cognitive impairment in older age. Clin Epidemiol.

[52] Chaudhuri J, Bains Y, Guha S (2018). The role of advanced glycation end products in aging and metabolic diseases: bridging association and causality. Cell Metab.

[53] Pérez MJ, Quintanilla RA (2015). Therapeutic actions of the thiazolidinediones in Alzheimer's disease. PPAR Res.

[54] Ahmed M, Cerda I, Maloof M (2023). Breaking the vicious cycle: the interplay between loneliness, metabolic illness, and mental health. Front Psychiatry.

[55] Kilgour AH, Rutherford M, Higson J (2024). Barriers and motivators to undertaking physical activity in adults over 70-a systematic review of the quantitative literature. Age Ageing.

[56] Phillips MC (2019). Fasting as a therapy in neurological disease. Nutrients.

[57] Varady KA, Bhutani S, Klempel MC (2013). Alternate day fasting for weight loss in normal weight and overweight subjects: a randomized controlled trial. Nutr J.

[58] Tinsley GM, La Bounty PM (2015). Effects of intermittent fasting on body composition and clinical health markers in humans. Nutr Rev.

[59] Wu P, Shen Q, Dong S, Xu Z, Tsien JZ, Hu Y (2008). Calorie restriction ameliorates neurodegenerative phenotypes in forebrain-specific presenilin-1 and presenilin-2 double knockout mice. Neurobiol Aging.

[60] Draicchio F, Axen KV (2025). Does energy restriction and loss of body fat account for the effect of intermittent fasting on cognitive function?. Nutrients.

[61] Mattison JA, Colman RJ, Beasley TM (2017). Caloric restriction improves health and survival of rhesus monkeys. Nat Commun.

[62] Willette AA, Bendlin BB, Colman RJ (2012). Calorie restriction reduces the influence of glucoregulatory dysfunction on regional brain volume in aged rhesus monkeys. Diabetes.

[63] Ching TT, Hsu AL (2025). The impacts of different dietary restriction regimens on aging and longevity: from yeast to humans. J Biomed Sci.

[64] Kraus WE, Bhapkar M, Huffman KM (2019). 2 years of calorie restriction and cardiometabolic risk (CALERIE): exploratory outcomes of a multicentre, phase 2, randomised controlled trial. Lancet Diabetes Endocrinol.

[65] Pali DV, Kim S, Mantik KE (2025). Unraveling the translational relevance of β-hydroxybutyrate as an intermediate metabolite and signaling molecule. Int J Mol Sci.

[66] Pawłowska M, Kruszka J, Porzych M, Garbarek J, Nuszkiewicz J (2025). Ketogenic metabolism in neurodegenerative diseases: mechanisms of action and therapeutic potential. Metabolites.

[67] Yancy WS Jr, Foy M, Chalecki AM, Vernon MC, Westman EC (2005). A low-carbohydrate, ketogenic diet to treat type 2 diabetes. Nutr Metab (Lond).

[68] Barrientos RM, Kitt MM, Watkins LR, Maier SF (2015). Neuroinflammation in the normal aging hippocampus. Neuroscience.

[69] Papenberg G, Ferencz B, Mangialasche F (2016). Physical activity and inflammation: effects on gray-matter volume and cognitive decline in aging. Hum Brain Mapp.

[70] Bjørklund G, Chirumbolo S (2017). Role of oxidative stress and antioxidants in daily nutrition and human health. Nutrition.

[71] Féart C, Samieri C, Barberger-Gateau P (2010). Mediterranean diet and cognitive function in older adults. Curr Opin Clin Nutr Metab Care.

[72] Fekete M, Varga P, Ungvari Z (2025). The role of the Mediterranean diet in reducing the risk of cognitive impairement, dementia, and Alzheimer's disease: a meta-analysis. Geroscience.

[73] Nie RZ, Luo HM, Liu YP (2024). Food functional factors in Alzheimer's disease intervention: current research progress. Nutrients.

[74] de Lima EP, Laurindo LF, Catharin VC (2025). Polyphenols, alkaloids, and terpenoids against neurodegeneration: evaluating the neuroprotective effects of phytocompounds through a comprehensive review of the current evidence. Metabolites.

[75] Schirinzi T, Martella G, Imbriani P (2019). Dietary vitamin E as a protective factor for Parkinson's disease: clinical and experimental evidence. Front Neurol.

[76] Shen L, Ji HF (2012). Vitamin E: supplement versus diet in neurodegenerative diseases. Trends Mol Med.

[77] Basambombo LL, Carmichael PH, Côté S, Laurin D (2017). Use of Vitamin E and C supplements for the prevention of cognitive decline. Ann Pharmacother.

[78] Miller ER 3rd, Pastor-Barriuso R, Dalal D, Riemersma RA, Appel LJ, Guallar E (2005). Meta-analysis: high-dosage vitamin E supplementation may increase all-cause mortality. Ann Intern Med.

[79] Zupo R, Castellana F, Panza F (2025). Alzheimer's disease may benefit from olive oil polyphenols: a systematic review on preclinical evidence supporting the effect of oleocanthal on amyloid-β load. Curr Neuropharmacol.

[80] Fiore M, Terracina S, Ferraguti G (2025). Brain neurotrophins and plant polyphenols: a powerful connection. Molecules.

[81] Hussain T, Tan B, Yin Y, Blachier F, Tossou MC, Rahu N (2016). Oxidative stress and inflammation: what polyphenols can do for us?. Oxid Med Cell Longev.

[82] Vauzour D, Vafeiadou K, Rodriguez-Mateos A, Rendeiro C, Spencer JP (2008). The neuroprotective potential of flavonoids: a multiplicity of effects. Genes Nutr.

[83] Sundaram JR, Poore CP, Sulaimee NH (2017). Curcumin ameliorates neuroinflammation, neurodegeneration, and memory deficits in p25 transgenic mouse model that bears hallmarks of Alzheimer's disease. J Alzheimers Dis.

[84] Gerenu G, Liu K, Chojnacki JE (2015). Curcumin/melatonin hybrid 5-(4-hydroxy-phenyl)-3-oxo-pentanoic acid [2-(5-methoxy-1H-indol-3-yl)-ethyl]-amide ameliorates AD-like pathology in the APP/PS1 mouse model. ACS Chem Neurosci.

[85] Vercambre MN, Berr C, Ritchie K, Kang JH (2013). Caffeine and cognitive decline in elderly women at high vascular risk. J Alzheimers Dis.

[86] Tiwari V, Mishra A, Singh S, Shukla S (2023). Caffeine improves memory and cognition via modulating neural progenitor cell survival and decreasing oxidative stress in Alzheimer's rat model. Curr Alzheimer Res.

[87] Chen JQ, Scheltens P, Groot C, Ossenkoppele R (2020). Associations between caffeine consumption, cognitive decline, and dementia: a systematic review. J Alzheimers Dis.

[88] Esposito S, Gialluisi A, Di Castelnuovo A (2024). Ultra-processed food consumption is associated with the acceleration of biological aging in the Moli-sani Study. Am J Clin Nutr.

[89] Cao L, Morley JE (2016). Sarcopenia is recognized as an independent condition by an International Classification of Disease, Tenth Revision, Clinical Modification (ICD-10-CM) code. J Am Med Dir Assoc.

[90] Kim D, Morikawa S, Miyawaki M, Nakagawa T, Ogawa S, Kase Y (2025). Sarcopenia prevention in older adults: effectiveness and limitations of non-pharmacological interventions. Osteoporos Sarcopenia.

[91] Chen H, Huang X, Dong M, Wen S, Zhou L, Yuan X (2023). The association between sarcopenia and diabetes: from pathophysiology mechanism to therapeutic strategy. Diabetes Metab Syndr Obes.

[92] Mesinovic J, Fyfe JJ, Talevski J (2023). Type 2 diabetes mellitus and sarcopenia as comorbid chronic diseases in older adults: established and emerging treatments and therapies. Diabetes Metab J.

[93] Wanagat J, Musci R, Herbst A, Aiken JM (2025). Mitochondrial DNA deletion mutations: a molecular cause of age-induced skeletal muscle fiber dysfunction and fiber death contributing to sarcopenia. Adv Exp Med Biol.

[94] Tseng BY, Gundapuneedi T, Khan MA (2013). White matter integrity in physically fit older adults. Neuroimage.

[95] Maass A, Düzel S, Goerke M (2015). Vascular hippocampal plasticity after aerobic exercise in older adults. Mol Psychiatry.

[96] Northey JM, Cherbuin N, Pumpa KL, Smee DJ, Rattray B (2018). Exercise interventions for cognitive function in adults older than 50: a systematic review with meta-analysis. Br J Sports Med.

[97] Dhahbi W, Briki W, Heissel A (2025). Physical activity to counter age-related cognitive decline: benefits of aerobic, resistance, and combined training-a narrative review. Sports Med Open.

[98] Saraulli D, Costanzi M, Mastrorilli V, Farioli-Vecchioli S (2017). The long run: neuroprotective effects of physical exercise on adult neurogenesis from youth to old age. Curr Neuropharmacol.

[99] Phillips C (2017). Lifestyle modulators of neuroplasticity: how physical activity, mental engagement, and diet promote cognitive health during aging. Neural Plast.

[100] Bherer L (2015). Cognitive plasticity in older adults: effects of cognitive training and physical exercise. Ann N Y Acad Sci.

[101] Yang TT, Lo CP, Tsai PS (2015). Aging and exercise affect hippocampal neurogenesis via different mechanisms. PLoS One.

[102] Lin TW, Shih YH, Chen SJ (2015). Running exercise delays neurodegeneration in amygdala and hippocampus of Alzheimer's disease (APP/PS1) transgenic mice. Neurobiol Learn Mem.

[103] Bolz L, Heigele S, Bischofberger J (2015). Running improves pattern separation during novel object recognition. Brain Plast.

[104] Trofin DM, Sardaru DP, Trofin D (2025). Oxidative stress in brain function. Antioxidants (Basel).

[105] Zhao H, Cheng R, Song G (2022). The effect of resistance training on the rehabilitation of elderly patients with sarcopenia: a meta-analysis. Int J Environ Res Public Health.

[106] Govindasamy K, Rao CR, Chandrasekaran B, Parpa K, Granacher U (2025). Effects of resistance training on sarcopenia risk among healthy older adults: a scoping review of physiological mechanisms. Life (Basel).

[107] Hurst C, Robinson SM, Witham MD (2022). Resistance exercise as a treatment for sarcopenia: prescription and delivery. Age Ageing.

[108] Izquierdo M, de Souto Barreto P, Arai H (2025). Global consensus on optimal exercise recommendations for enhancing healthy longevity in older adults (ICFSR). J Nutr Health Aging.

[109] Xu J, Wan CS, Ktoris K, Reijnierse EM, Maier AB (2022). Sarcopenia is associated with mortality in adults: a systematic review and meta-analysis. Gerontology.

